# Factors associated with cancer-related fatigue in breast cancer patients undergoing endocrine therapy in an urban setting: a cross-sectional study

**DOI:** 10.1186/1471-2407-10-453

**Published:** 2010-08-23

**Authors:** Xu Huang, Qingyuan Zhang, Xinmei Kang, Ying Song, Wenhui Zhao

**Affiliations:** 1 Department of Medical Oncology, Tumor Hospital of Harbin Medical University, Harbin, China

## Abstract

**Background:**

Fatigue is prevalent in breast cancer survivors and has profound effects on daily life. The interference of fatigue with endocrine therapy may be difficult to separate. This study investigates the prevalence and severity of fatigue and identifies the demographic, clinical, and lifestyle factors associated with cancer-related fatigue (CRF) in breast cancer patients undergoing endocrine therapy in an urban area.

**Methods:**

Women with stage I-IIIA breast cancer were recruited and asked to participate (n = 371) in the study. The 315 women who responded to the questionnaire (84.9%), 54 (17.1%) had completed endocrine therapy and 261 (82.9%) were still undergoing endocrine therapy. The patients had been diagnosed at an average of 31 months prior to recruitment (range, 7 to 60 months); the average age was 48 (range, 33 to 72) years. The 11-point scale and Visual Analog Scale (VAS) were employed to quantify the level of fatigue experienced by the patients. Logistic regression analyses and a trend test method were performed to evaluate factors associated with CRF.

**Results:**

Among the 315 patients, 189 (60%) had experienced or were experiencing CRF during endocrine therapy. Logistic regression analysis was performed to identify factors associated with CRF, including BMI (body mass index), clinical stage, menopausal status, duration of endocrine therapy, physical activity, and diet. Factors unrelated to CRF were age, marital status, treatment, endocrine therapy drugs, alcohol intake, and smoking. The trend test method revealed an association between physical activity and dietary level and the intensity of CRF.

**Conclusions:**

The present findings suggest that fatigue is an important problem in the majority of breast cancer patients during endocrine therapy. We found that BMI, clinical stage, menopausal status, duration of endocrine therapy, physical activity, and diet are associated with fatigue. Future research should focus on the impact factors of CRF and lifestyle in the management of breast cancer patients.

## Background

Cancer survivors comprise a vulnerable population that has distinct therapy needs. A large proportion of cancer patients experience cancer treatment-related physical and mental symptoms. Fatigue is the most frequently reported symptom among cancer patients [1-6], with an estimated 60-96% of cancer patients who are undergoing treatment experiencing fatigue, including 60-93% of radiotherapy and 80-96% chemotherapy patients [5-7]. Cancer-related fatigue (CRF) is a distressing, persistent, and subjective sense of physical, emotional and/or cognitive tiredness or exhaustion related to cancer or cancer treatment that is not proportional to recent activity and interferes with normal functioning [8]. Compared with the fatigue experienced by healthy individuals, CRF is more severe, more distressing [9,10], and is less likely to be relieved by rest [11]. Patterns of the occurrence of fatigue may differ according to the type of treatment [12] and the stage of cancer [13].

Breast cancer is notable for causing long-term treatment effects that begin during the treatment period and continue after therapy [14]. Certain symptoms are more prominent in women with breast cancer, including fatigue, hot flashes, sexual dysfunction, infertility, bone loss, and cognitive dysfunction [15]. Studies indicate that the prevalence of fatigue is high in patients with breast cancer; as many as 99% of these patients experience fatigue during the course of treatment [16]. CRF could be mediated by the endocrine sequelae of breast cancer treatment, including the induction of amenorrhea, the induction of premature menopause due to ovarian toxicity caused by chemotherapy, radiotherapy and the side effects of adjuvant endocrine treatment [17]. It has been proposed that hormone treatment has minimal effects on fatigue and menopausal symptoms. However, adjuvant chemotherapy leads to severe fatigue and menopausal symptoms during and after treatment, and worse symptoms are observed with chemotherapy-induced treatment than with natural menopause [18]. The interference of fatigue with menopausal symptoms, however, may be difficult to elucidate, especially in young breast cancer patients who face premature and sudden menopause and prolonged oestrogen deprivation.

Endocrine therapy was proposed as a treatment for breast cancer, especially in patients who had undergone premature menopause and in post-menopausal women with hormone-responsive, oestrogen receptor-positive tumors. Fatigue in breast cancer survivors may be more distressing than fatigue in the general population, because many women experience premature menopause due to chemotherapy or oestrogen deprivation treatment and thus experience menopause-related fatigue more abruptly and earlier in life than do those with a more natural menopausal transition [14,15]. Gélinas C et al. also showed that persistent fatigue in younger breast cancer patients following the completion of therapy was related to menopausal symptoms [19].

Activity enhancement and psychosocial interventions are two nonpharmacologic interventions with strong evidence for the treatment of fatigue. Regular exercise leads to a decrease in fatigue, depression, and anxiety both during [20-22] and after [23-26] cancer treatment in breast cancer patients. However, there is also some evidence that dietary management and sleep therapy can relieve fatigue symptoms [27].

Thus, it can be hypothesised that menopausal status and therapies in patients with breast cancer may participate in the development of fatigue. In contrast to fatigue during chemotherapy and radiotherapy, fatigue during endocrine therapy in women with breast cancer has been reported less frequently. We hypothesised that some demographic and clinical factors, such as age, BMI (body mass index), clinical stage, menopausal status, and duration of endocrine therapy, are associated with CRF in breast cancer during endocrine therapy. We also hypothesised that some effective nonpharmacologic interventions, such as physical activity and diet, could improve CRF.

## Methods

### Participants

A cross-sectional study was performed to assess women living with breast cancer who had completed or were undergoing endocrine therapy in an urban area. These patients were diagnosed with breast cancer and/or received treatment for breast cancer at the Tumor Hospital of Harbin Medical University, Harbin, China, between June 2004 and September 2009. Patients had been diagnosed at an average of 31 months prior to recruitment (range, 7 to 60 months); the average age was 48 (range, 33 to 72) years. All cases were confirmed by a pathological examination; oestrogen receptor (ER) and progesterone receptor (PR) statuses were evaluated by standard immunohistochemistry. All patients were ER-positive and PR-positive or PR-negative. The eligibility criteria for study participation were as follows: a) at least 18 years of age, b) no documented or observable psychiatric or neurological disorders that would interfere with participation, c) women diagnosed with stage I-IIIA breast cancer, d) no history of another cancer other than basal cell skin carcinoma, e) no other chronic or life-threatening diseases in which fatigue is a prominent symptom (e.g., multiple sclerosis, rheumatoid arthritis, fibromyalgia, or chronic fatigue syndrome), f) previous surgical treatment for breast cancer, g) completed or undergoing endocrine therapy, h) endocrine therapy for more than six months, and i) provision of written informed consent.

### Questionnaires Procedures

Recruitment strategies included interviews with women who were identified from tumor registries and referrals from clinical oncologists. Eligibility was determined based on a chart review and consultation with the attending physician. One of these women was randomly selected and sent a letter of introduction. Three hundred and seventy-one women were considered eligible and asked to participate in the study, and 333 accepted. Those women who provided informed consent were instructed to complete a series of questionnaires that assessed factors associated with CRF. In total, 315 women responded to the questionnaire (Additional file 1) (84.9%). Among the 315 patients, 54 patients (17.1%) had completed endocrine therapy, 261 patients (82.9%) were undergoing endocrine therapy, and the incidence of premature menopause was 27.6%. The remaining patients were excluded because a complete data set was not available due to inaccurate filing and missing or unavailable records. The address, phone number, and disease status of each patient was confirmed, and the treating physician was contacted for permission to contact the patient. For some patients, endocrine therapy had been initiated five years prior, but other patients were still undergoing endocrine therapy. However, our hospital had a regular follow-up system and documentation from the first admission to death. These records helped the two groups of patients recall their conditions, thereby improving the accuracy of the questionnaire contents and avoiding bias.

The survey, which was designed to be self-reported, included items that provided the following information: age, BMI, marital status, alcohol intake, smoking, menopausal status, clinical stage, endocrine therapy status, CRF degree, physical activity level, and dietary assessment. The ethics committee of Tumor Hospital of Harbin Medical University approved the study.

### Questionnaires

#### Cancer-related fatigue levels

The patients were screened for fatigue using the 11-point scale [28] (0 = not fatigued, 10 = extremely fatigued) and the Visual Analog Scale (VAS) [29,30], which assessed the level of fatigue among the respondents. The fatigue scale for adults was based on a 0-10 point scale. Using the scale, fatigue was divided into the following categories: none (0 points), mild (1-3 points), moderate (4-6 points), and severe (7-10 points). Clinical experience with the assessment of fatigue has shown that some patients cannot define their fatigue based on a numeric value. This small group of patients rated their fatigue on the VAS. VAS is frequently used to assess subjective feelings such as pain and fatigue [29]. The answer line of 100 mm ranges from "not at all fatigued" to "as fatigued as I could be." VAS was divided into three parts of equal, and fatigue was classified into categories as follows: none (0 mm), mild, moderate, or severe. Consequently, all of the patients classified fatigue as mild, moderate, or severe. The respondents indicated whether their fatigue had changed during endocrine therapy, and fatigue was assessed when the participant was completing the questionnaire.

#### Physical activity levels

The intensity, duration, and frequency of physical activity were self-reported by the participants and converted into metabolic equivalents (METs). Activity choices included the following: walking or hiking outdoors, jogging (> 10 minutes per mile), running (≤ 10 minutes per mile), biking (including stationary bike), swimming laps, tennis, callisthenics, aerobics, aerobic dance, rowing machine, among others. Qigong and Tai Chi Chuan were both considered types of aerobic exercise. Levels of general physical activity and walking were assessed separately and combined to achieve a measurement of total METs. Total energy expenditure was determined by analyzing the time (minutes) spent on physical activity per week by METs; it was then divided into the following categories: light physical activity (< 3 METs), moderate activity (3-6 METs), and vigorous activity (> 6 METs). Walking was assigned a value of 2, 3, 4 or 6 METs based on the walking speed, which was characterised as slow, average, fast, or very fast [31]. Total METs were calculated by summing the METs determined for each category of activity and multiplying the result by the duration in minutes. The MET-hours/week (MHW) was then calculated by dividing the total METs by 60 [32]. Physical activity was assessed and categorised based on the MET task hours per week (MHW) as follows: 3.3≤ MHW < 10, 10≤ MHW < 20, MHW ≥ 20.

#### Dietary assessments

Typical data for dietary intakes were obtained via the participants' self-reports. According to the Dietary Guidelines For Chinese Residents (2007), the daily intake of an average person should be as follows: A. cereals, 250-400 g; B. vegetables and fruits, 300-500 g and 200-400 g, respectively; C. fish and shrimp, 50 g; poultry and meat, 50-100 g; eggs, 25-50 g; D. milk and milk products, 100 g; beans and bean products, 30-50 g; E. fats and oil, less than 25 g. The levels of these nutrients were calculated according to the intake of A to E; each category represented 1 point. Scores were added up to yield a final score that ranged from 0 (met none of the recommendations) to 5 (met all of the recommendations). The dietary assessment was categorised as follows: 0, 1-2, 3-4, and 5.

### Statistical analysis

All analyses were based on data obtained from the aforementioned questionnaires, which were certified by clinical oncologists and by the participants themselves. Multiple logistic regression analyses were employed to determine factors associated with CRF in breast cancer patients undergoing endocrine treatment. The trend test method was used to evaluate the association between physical activity and dietary level and the intensity of CRF. All statistical analyses were performed using SPSS 13.0 software.

## Results

Among the three hundred and fifteen patients who responded to the questionnaire, 189 (60%) experienced or were experiencing CRF during endocrine therapy. Table 1 illustrates the demographic data for the patients that were included in the study. Among the 315 patients, those with mild, moderate, and severe fatigue comprised 44.8%, 9.8% and 5.4% of the study population, respectively. Within these 189 patients, 173 (91.5%) had reduced CRF, whereas 16 (8.5%) had the same or increased fatigue. Logistic regression analysis was performed to identify factors that were associated with CRF. As shown in Table 2, the significant factors associated with CRF included BMI, clinical stage of the tumor, menopausal status, duration of endocrine therapy, physical activity, and diet. Age, marital status, treatment types, drugs used for endocrine therapy, alcohol intake, and smoking did not affect CRF.

**Table 1:** Information of Subjects(Total 315 patients)

Category	Number and percentage
	**N(%)**

1. Age(year)	
30--49	156(49.4)
50--64	102(32.4)
≥ 65	57(18.2)
2. Body mass index(kg/m^2^)^a^	
≤ 25 kg/m^2^	240(76.1)
> 25 kg/m^2^	75(23.9)
3. Marital status	
Married	293(93.0)
Single	22(7.0)
4. Menopausal status	
Pre-menopause	113(36.0)
Post-menopause	202(64.0)
5. Clinical stage	
Stage I	72(22.9)
Stage II	135(42.9)
Stage IIIA	108(34.3)
6. Treatment	
Surgery + endocrine therapy	21(6.7)
Surgery + radiation + endocrine therapy	48(15.2)
Surgery + chemotherapy + endocrine therapy	91(28.9)
Surgery + chemotherapy + radiation + endocrine therapy	155(49.2)
7. Drugs for endocrine therapy	
Pre-menopause	
Tamoxifen	143(45.4)
Post-Menopause	
Tamoxifen	38(12.1)
Aromatase inhibitors	134(42.5)
8. Duration of endocrine therapy	
> 6 Months to 3 years	173(54.9)
> 3 years to 5 years	142(45.1)
9. Alcohol intake	
yes	61(19.4)
no	254(80.6)
10. Smoking	
yes	4(1.3)
no	311(98.7)

a. Body mass index (BMI): BMI > 25 kg/m^2 ^indicates overweight;

BMI > 30 kg/m^2 ^indicates obese.

**Table 2:** Logistic Regression Analysis to Identify Factors Associated with CRF

	95% CLs			
	**Odds Ratio**	**Lower**	**Upper**	**P**

Body mass index	3.200	0.959	10.684	0.0586
Cinical stage	11.720	1.331	103.212	0.0266
Menopausal status	7.331	1.531	35.099	0.0127
Duration of endocrine therapy	5.491	1.043	28.901	0.0444
Physical activity	2.765	0.960	7.962	0.0595
Diet	0.242	0.097	0.605	0.0024

In the 173 patients who showed a reduced level of fatigue, a correlation was detected between physical activity and dietary level and the intensity of CRF, as demonstrated in Table 3. Those patients with a reduced level of CRF showed the following CRF intensity and mean physical activity level: mild CRF/15.0 MHW; moderate CRF/11.5 MHW; severe CRF/6.7 MHW. The average physical activity level of patients with reduced CRF was 823 METs/week (or 13.7 MHW). The trend test method showed that the intensity of CRF decreased with an increase in physical activity (p < 0.05). Among these patients who had reduced CRF, 43.9% and 24.9% of the patients performed Qigong and Tai Chi, respectively, and 11.2% performed both Qigong and Tai Chi. A total of 83.7% of the patients with mild relievable CRF and 59.6% of the patients with moderate relievable CRF demonstrated an active or a highly active physical activity level (10.0 ≤ MHW < 20.0 or MHW ≥ 20.0). Among the patients who showed relieved CRF, approximately 45.7% achieved 5 points in the dietary assessment. Another 27.5%, 11.8%, 8.7%, 5.2% and 1.2% of patients demonstrated dietary levels of 4, 3, 2, 1 and 0, respectively. Approximately 81.1% of the patients obtained 60% of their total calories from cereals, 71.3% obtained 15-20% from proteins and fats, and 61.8% consumed a satisfactory amount of fruits and vegetables. The trend test method showed that the intensity of CRF decreased with an increase in the dietary level (p < 0.05).

**Table 3:** The correlation between the intensity of CRF and the level of physical activity and diet in patients with relieved CRF

	Intensity of Fatigue						
	**Mild**		**Moderate**		**Severe**		**P**

	**N**	**%**	**N**	**%**	**N**	**%**	

1. Physical Activity							< 0.0001
MHW < 3.3	0	0	1	2.7	3	42.9	
3.3≤ MHW < 10.0	21	16.3	14	37.8	2	28.6	
10.0≤ MHW < 20.0	88	68.2	16	43.2	2	28.6	
MHW ≥ 20.0	20	15.5	6	16.2	0	0	
2. Diet							0.02
0	0	0	1	2.7	1	14.3	
1~2	16	12.4	6	16.2	2	28.6	
3~4	52	40.3	13	35.1	3	42.9	
5	61	47.3	17	45.9	1	14.3	

Statistically significant (p < 0.05)

## Discussion

The impact of CRF on quality of life and the ability to carry out activities of daily living have profound implications for patients, especially those who experience a noticeable decline in their functional status while undergoing cancer treatment [1,33]. It has been reported that the side effects of hormone cancer treatment or hormone deprivation may unduly impair quality of life [34,35]. In addition to hormone cancer treatment and hormone deprivation, CRF has been associated with the increased production of different cytokines, increased tumor growth, impaired energy transformation processes and many other cancer-related pathologies [16,36]. The phenomenon of CRF is complex and multifactorial and is currently not fully understood.

In the present study, 60% of the patients were experiencing or had experienced CRF during endocrine therapy. These findings support the available evidence that occurrence rates for fatigue are generally higher in other cancer populations than in breast cancer patients [36]. Malinovsky et al. suggested that different levels of fatigue were associated with different groups of anti-hormones [37]. However, the type of endocrine therapy drug used was not associated with fatigue in our study. This finding may suggest that CRF is not associated with anti-oestrogens, which are used to treat breast cancer patients.

We found that BMI correlated with CRF and that 23.9% of the patients were overweight. BMI tends to increase in women who have undergone breast cancer treatment [38], due to changes in hormone levels [39], altered eating behaviours [40], and other psychological factors [41]. Rock CL et al. suggested that obesity was a side effect of endocrine therapy [42]. To achieve a balance between adequate nutrition and energy consumption, professional interventions such as a combination of diet changes and physical activity may be more effective [43-45]. Goodwin et al. suggested that an evaluation of diet and physical activity should be conducted within one year after the diagnosis of early stage breast cancer [46].

In our study, the degree of CRF was further correlated with the clinical stage, the menopausal status and duration of endocrine therapy. The pre-menopausal patients showed a greater tendency toward CRF during the first 3 years of the endocrine therapy. The early detection of fatigue will provide important information to guide the clinical management of CRF, such as the timing [13] and the types of treatment [12]. CRF should be screened and evaluated according to clinical guidelines to provide patients with an accurate consultation and appropriate management.

Among the non-pharmacologic interventions, exercise showed the strongest evidence for a positive effect in the management of CRF [43,44,47,48]. Physical activity has been beneficial in improving symptoms of fatigue in breast cancer survivors [49-52]. In the present study, 61.3% of the patients who reduced CRF demonstrated active physical activity levels. This result might be due to the finding that most of the patients suffered from mild CRF (74.6%); they could perform some higher than moderate levels of exercise. Traditional Chinese sports games such as Qigong and Tai Chi are typical aerobic activities and are considered high-level physical activities (> 6 METs), which is above the suggested physical activity level. Current recommendations for exercise among cancer patients are walking at a moderate pace for up to 30 min/day (slowly building up to 30 min/day) three or more times per week [15]. Moderate rather than vigorous exercise is preferable for patients and results in increased compliance [53]. Further investigation is required to determine whether the present results are applicable to breast cancer patients with CRF.

Regarding the effects of diet on CRF, we found that a greater number of points for diet correlated with a lower CRF intensity. Among the patients with relieved CRF, 72.3% demonstrated a dietary level of 4 or 5. Thus, we can speculate that most cancer patients in large and medium-sized cities have a satisfactory diet with balanced nutrition. Other studies have shown that multiprofessional patient management is critical for patient recovery, including dietary counselling, nutrition intervention, and medicine [1,54]. Physical activities can improve appetite, relieve constipation [54], and maintain sufficient nutritional stores, which will certainly benefit the health of the patient [3,55].

## Limitations

The results of this study reflect the retrospective nature of the self-reported data, and they may demonstrate a potential recall bias. However, during endocrine treatment of breast cancer in women, this limitation of the data also provides significant information. The data show the prevalence and severity of fatigue, as well as the demographic, clinical, and lifestyle factors associated with CRF in breast cancer patients undergoing endocrine treatment in an urban area.

## Conclusions

The present findings suggest that fatigue is an important problem in the majority of breast cancer patients during endocrine therapy. We found that BMI, clinical stage, menopausal status, duration of endocrine therapy, physical activity, and diet are associated with fatigue. Future research should focus on the impact factors of CRF and lifestyle in the management of breast cancer patients.

## Competing interests

The authors declare that they have no competing interests.

## Authors' contributions

XH was the main author of the manuscript, participated in concept, design, data analysis, data interpretation and writing. QZ participated in concept, design, statistical analysis, and data interpretation. XK, YS and WZ participated in statistical analysis, and data interpretation. All authors read and approved the final manuscript.

## Pre-publication history

The pre-publication history for this paper can be accessed here:

http://www.biomedcentral.com/1471-2407/10/453/prepub

## Supplementary Material

Additional file 1 **Questionnaire (Breast Cancer)**. This questionnaire was used to investigate factors associated with cancer-related fatigue among breast cancer during endocrine treatment in an urban area. Click here for file
