# Supplementary material for: Factors associated with cancer-related fatigue in breast cancer patients undergoing endocrine therapy in an urban setting: a cross-sectional study
Source: BMC Cancer. 2010 Aug 23;10:453. doi: 10.1186/1471-2407-10-453 (PMC2939549; doi:10.1186/1471-2407-10-453)
Supplement: Additional file 1 — Questionnaire (Breast Cancer). This questionnaire was used to investigate factors associated with cancer-related fatigue among breast cancer during endocrine treatment in an urban area. [file 1471-2407-10-453-S1.DOC]

**Additional files**

**Additional file 1**

**Title:** **Questionnaire (Breast Cancer)**

**Description:** This questionnaire was used to investigate factors associated with cancer-related fatigue among breast cancer during endocrine treatment in an urban area.

**QUESTIONNAIRE**

(BREAST CANCER)

Department of Medical Oncology,

Tumor Hospital of Harbin Medical University, Harbin, China

Telephone: (0451)86298673

http://www.hrbmutumour.cn

This questionnaire is to investigate factors associated with cancer related fatigue among breast cancer during endocrine treatment. Please carefully read the questionnaire and complete it under the guidance of a doctor. Thank you for your support!

**Part 1**
Name: Date of birth:

Today’s date

Height: Weight:

Chest circumference: Waistline:

Hip circumference:
Pathologic diagnosis:

Estrogen receptor (ER):

Progesterone receptor (PR):

HER-2(cerbB-2):

Telephone：home Work

【1】 Marital status:
 ①Married ② Unmarried
【2】 Drinking:
 ①Occasionally ②Regular ③Never
【3】 Smoking:
 ①Occasionally ②Frequent ③Never
【4】The first time treatment method was :
 ①Operation ②Chemotherapy ③Radiotherapy ④Chinese medicine treatment ⑤ Endocrine therapy ⑥Other (please write)
【5】Did you experience surgery?
    ① Yes ② No
【6】Did you experience breast-conserving surgery?
 ① Yes ② No
【7】Did you experience chemotherapy?
 ① Yes ② No
【8】 Did you experience radiotherapy?
     ① Yes ② No

**Part 2**
【1】 Are you already in the state of post-menopausal?

     ① Yes ② No
【2】 What kind of drug did you take during endocrine therapy?(one or two)
     ①Tamoxifen ②Anastrozole ③Toremifene ④ Letrozole
【3】How long have you been during endocrine therapy ?
    ①Between more than six months and three years

②Between more than three years and five years
【4】 The most obvious discomfort during endocrine therapy was
【5】 Did your body weight increase during endocrine therapy? kg
    ① Yes ② No
【6】What kind of your mood was during endocrine therapy?
 ①Smooth ②Depression ③Anxiety ④ Happiness
【7】Do you have or have you ever had bone and joint pain during endocrine therapy?

     ① Yes ② No
【8】Do you have or have you ever take calcium ?
      ① Yes ② No
【9】What is the effect of calcium ?
     ① Bone and joint pain disappeared ②no mitigation
【10】Treatment of bone and joint pain in other ways, please write
    
**Part 3**
Current and prior dietary intake data were obtained via repeated 24-h dietary logs.

【1】What kind of meals was in your home ? (Please write the type and amount, the unit is g)
① Animal food main ②Vegetable diet main ③Equal meat and vegetables
【2】What kind of food was your breakfast ? (multiple choice) (Please write the intake, the unit is g)
① Soya bean milk or semi-congealed jellied bean curd ②Milk ③Eggs ④Rice, steamed bread, bread and other cereal products ⑤ Instant noodles, instant food ⑥ Deep-fried fritters, fried cake, pancakes and other fried foods

⑦Pickles ⑧Porridge ⑨Fruit ⑩ Drinks
【3】What kind of food do you often eat ? (Please write the intake, the unit is g)
①Instant noodles ② Sweetie ③Fried food ④Pickled food ⑤Canned food ⑥Ham sausage ⑦Puffed food ⑧Bacon food ⑨Water-fat seafood

⑩Ice cream
【4】Do you eat fruits and vegetables everyday? (Please write the type and intake, the unit is g)
① Fruits ② Vegetables
【5】How much water do you drink everyday? (500 ml is about 2 cups)
①less than a cup ② 1-2 cups ③ 3-4 cups ④ 5-6 cups ⑤ 7-8 Cups⑥ 8 or more cups ⑦ Very little water
【6】 Your daily drink is: (multiple choice)
① Coffee ②Tea ③Carbonated beverage ④Milk ⑤ Fruit juice ⑥ Milk beverages ⑦ Yogurt and lactic acid drinks ⑧Other functional drinks
【7】What frequency of your drinking the milk was (please write the type and intake, the unit is g)
①Everyday ②Sometimes ③Occasional ④Seldom ⑤ Never
【8】What frequency of your drinking the soy milk was (please write the type and intake, the unit is g)
①Everyday ②Sometimes ③Occasional ④Seldom ⑤ Never
【9】The main way of the cooking in your home was (selection of the most common first 2 species)
① Oil heating (saute frying) ②Water heating (steaming stew) ③ Electric heating (microwave, oven) ④ Without heating (cold, raw)
【10】what kind of food was your favorite taste? (multiple choice)
① local traditional cuisine taste ②Cantonese cuisine taste ③Sichuan cuisine taste ④Shandong cuisine taste ⑤ Jiangsu, Zhejiang and Shanghai cuisine taste ⑥ Japanese and Korean cuisine taste ⑦Northeast cuisine taste ⑧ Western cuisine taste ⑨Other taste ⑩ Arbitrary or not fixed
【11】 What kind of agricultural products do you often eat? (Please write the type and intake, the unit is g)
①Fish and shrimp ②Poultry and meat ③ Eggs

【12】what kind of lipid do you intake everyday? (please write the type and intake, the unit is g)
 ① From animal ② From plant

**Part 4**
【1】 The frequency of your exercise is (please write total time for activities)
    ① Never or occasionally ②Once a week ③ Two or three times a week ④ More than four times a week
【2】Your exercise is

 ① ball (table tennis, badminton, tennis, volleyball, basketball, bowling, football, billiards )
 ②Running ③Brisk walking ④Walking ⑤Tai chi ⑥Yoga ⑦Dancing ⑧Qigong (name ) ⑨ Others
【3】The time of your outdoor activities everyday was
 ①0.5-1 hours ②1-2 hours ③2 – 4 hours ④0.5 hours below ⑤4-8 hours ⑥8 hours or more
【4】 Your working hours everyday was
    ① 5 hours ② 5 - 8 hours ③ 8-10 hours ④ 10 hours or more
【5】 Your sleeping time everyday was
   ① More than 8 hours ② 6 ~ 8 hours ③ 4 ~ 6 hours ④ Less than 4 hours

【6】Sleeping time in the day time was
① More than 45 minutes ②Less than 45 minutes ③ Irregular ④Always want to go to bed

**Part V**
【1】 Do you have, or have you ever had experienced fatigue? (referring to a distressing, persistent, subjective sense of physical, emotional and/or cognitive tiredness or exhaustion, rest can not be relieved.)

① Yes ② No
【2】Fatigue evaluation is based on a 0-10 point scale (0=No fatigue; 10=Worst fatigue one can imagine).

Please write the score
【3】During endocrine therapy, fatigue score was (Please write the score)
①Reduce ② Increase
【4】Fatigue started (what time), the duration

(how long)
【5】Fatigue often Recurred

① Yes ② No
【6】Fatigue was persistent
 ① Yes ② No
【7】Fatigue was mitigated or eliminated by means of (multiple choice)
  ①Chemotherapy ②Radiotherapy ③Improving the treatment of immune system ④ Chinese medicine treatment ⑤Endocrine therapy ⑥ Moderate physical exercise ⑦Cultural activities ⑧Emotional adjustments ⑨Diet ⑩Other treatment of the diseases unrelated to tumor (please write )
【8】Do you have, or have you ever had, any of the following symptoms? (multiple choice)
①Pain ②Depression or anxiety ③Anemia ④Sleep disorders (insomnia or drowsiness)
⑤The change of weight, energy intake and nutritional status (decrease or increase, select one)
⑥ Activity amount (reduction or increase, select one), and physical status (reduced or enhanced, select one)

【9】Do you have, or have you ever had, the following diseases? (multiple choice)
① Lung disease ②Heart disease ③Liver disease ④Kidney disease ⑤Nervous system diseases ⑥Infected disease
⑦Endocrine system diseases (hypothyroidism, ovarian dysfunction, adrenal insufficiency) ⑧ bone and joint diseases
⑨Colonic diseases ⑩Pancreatic diseases
Other diseases (please write )
